# Resection of an Intramedullary Glomus-type Arteriovenous Malformation of the Conus Medullaris: Technical Note and Step-by-step Surgical Video

**DOI:** 10.7759/cureus.4022

**Published:** 2019-02-05

**Authors:** Salah G Aoun, Tarek Y El Ahmadieh, Aaron R Plitt, Jake Kreck, Kevin C Morrill

**Affiliations:** 1 Neurosurgery, University of Texas Southwestern Medical Center, Dallas, USA

**Keywords:** arteriovenous malformation, glomus arteriovenous malformation, conus, spine vascular malformation, surgical video, surgical education, surgical technique

## Abstract

Arteriovenous malformations (AVMs) of the spine include a broad spectrum of lesions that vary from a simple arteriovenous fistulous connection to a more complex net of abnormal vessels involving multiple spinal levels. These entities are poorly studied and understood because of their rarity and are often either managed conservatively with observation if the lesion is complex, or treated surgically or interventionally in the presence of an accessible and distinct fistulous connection. Most surgeons avoid intervening on more intricate lesions until they become symptomatic with progressive neurological decline.

We describe the case of a 38-year-old man who presented with severe sharp back pain after an appendectomy procedure. A magnetic resonance angiogram (MRA) revealed an arteriovenous malformation of the conus medullaris, with a compact glomus-type nidus and arterial feeders originating from an enlarged artery of Adamkiewicz. The malformation was resected through a posterior midline approach, and the patient was neurologically intact at his discharge on postoperative Day 2. Follow-up angiography showed complete obliteration of the lesion. Our operative video is meant to serve as a step-by-step and systematic guide to the approach and management of conus arteriovenous spinal lesions, which can be difficult to treat.

We provide a pre- and postoperative radiological description of the anomaly as well as a technical guide to the resection of a spinal vascular lesion. This video could serve as an operative guide and reference to neurosurgeons—both established and in training—when confronting similar disease processes in the future.

## Introduction

Arteriovenous malformations (AVMs) of the conus medullaris are rare entities whose natural history is defined by continued progression once they become symptomatic [1]. They usually have an intradural component, with or without dural and epidural vessels, and can present different degrees of parenchymal involvement [2]. The surgical management of these lesions can be difficult, and the stakes are high when the conus or the spinal cord are involved [3]. The aim of this report is to provide the reader with surgical pearls that can make resection easier, specifically for conus lesions, and with visual aid and support through a commented video case report.

## Technical report

**Figure 1:**
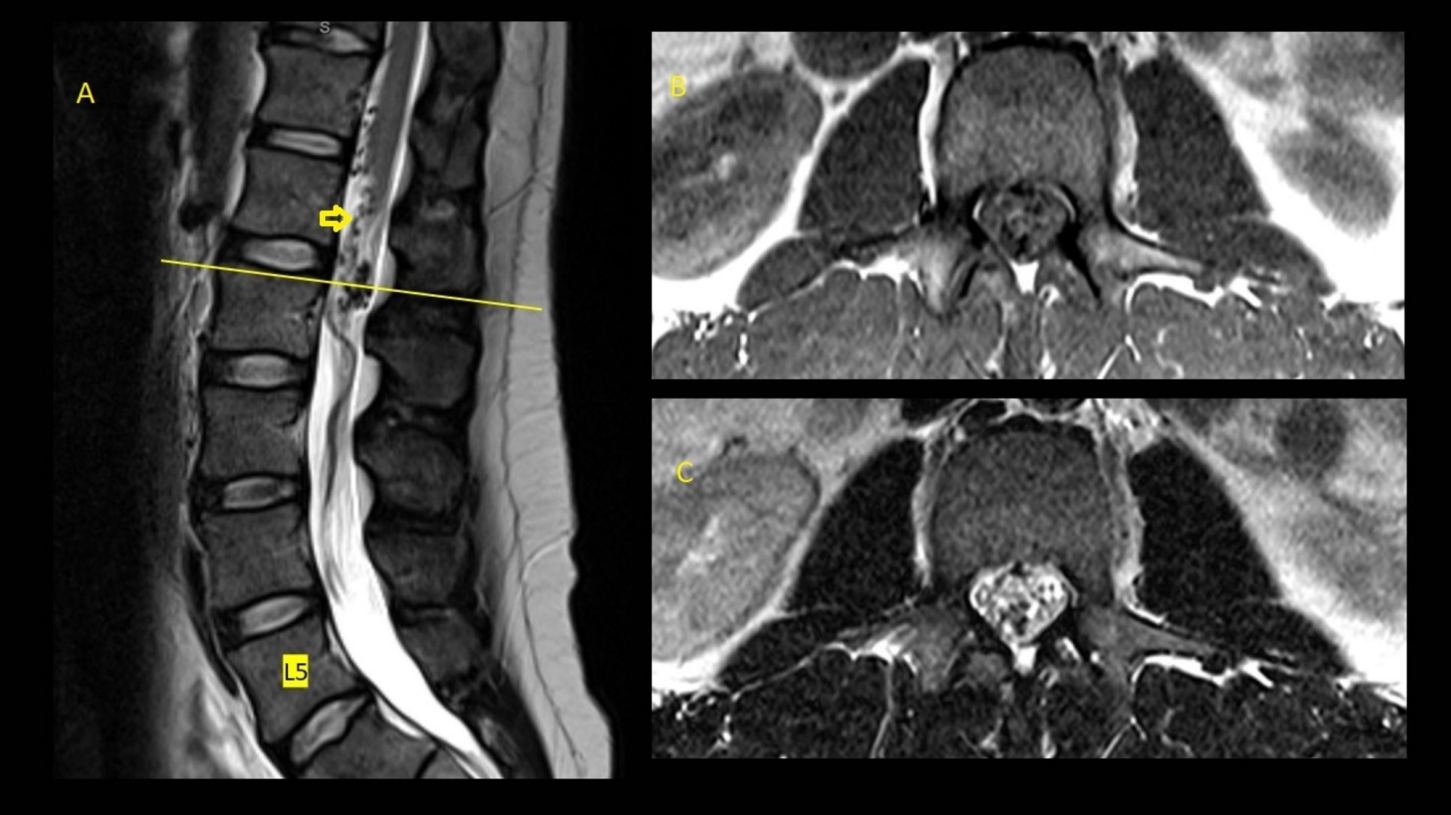
Sagittal T2 (A) and axial T1 (B) and T2 (C) magnetic resonance imaging showing the conus arteriovenous malformation with tortuous vessels surrounding the filum terminale. The yellow arrow points at the junction point between the filum and the vascular malformation.

**Figure 2:**
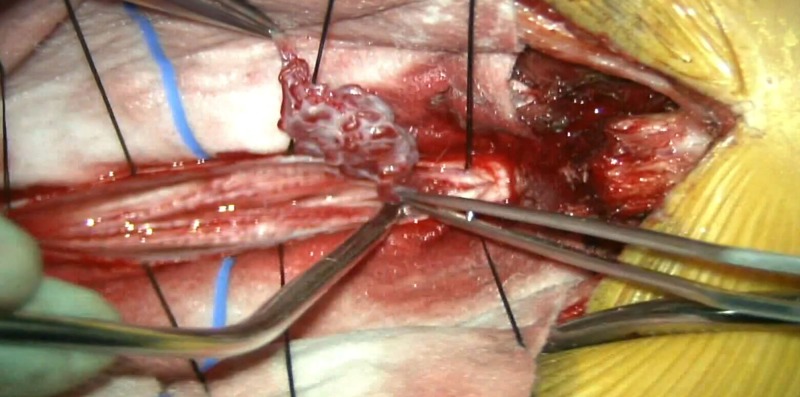
Intraoperative microscope image showing the vascular malformation fully dissected from the tip of the conus, immediately prior to the time of resection.

A 38-year-old man experienced sharp lower back pain after an appendectomy procedure. The pain was followed by tingling in the bilateral lower extremities and resolved quickly. A magnetic resonance angiogram (MRA) of the lumbar spine was obtained (Figure 1) and revealed abnormal vascular loops and flow voids at the level of the conus from T12 to L2. A catheter angiogram showed an enlarged artery of Adamkiewicz transitioning into an enlarged anterior spinal artery that was supplying the malformation (Video 1). The nidus was compact and looped around the conus. The decision was taken to surgically remove the malformation. The patient was taken to the operating room after appropriate counseling regarding the risks of the procedure, which included injury to the spinal cord, spinal cord infarction, cerebrospinal fluid leakage, and incomplete resection of the malformation. Neuromonitoring for somatosensory evoked potential, electromyogram, and motor stimulation of bilateral lower extremities as well as anal sphincter function was obtained. A midline incision was performed, with a laminectomy from T12 to L2, followed by a midline durotomy which revealed the arteriovenous malformation. An indocyanine green (ICG) angiogram was performed and showed the abnormal vessels. The arterial feeder flow appeared to go from rostral to caudal. One of the key maneuvers that allowed us to mobilize the lesion and free it from the conus was sectioning the filum terminale (Figure 2). The pial plane did not seem to go deep into the substance of the conus, and a temporary clip was placed on the arterial pedicle. This relaxed the lesion substantially and further facilitated its manipulation. Another ICG angiogram was obtained with the clip in place and showed that there was no arterial inflow in the malformation (Video 1). The pedicle was then coagulated and sectioned. The patient recovered well from the procedure, and a postoperative magnetic resonance angiogram showed no residual.

**Video 1:** Surgical technical video: Intraoperative video showcasing the resection of an arteriovenous malformation of the conus medullaris. This video also serves as a technical guide for the diagnosis and treatment of the described lesion.

## Discussion

Arteriovenous malformations of the conus can be challenging to treat especially for asymptomatic or mildly symptomatic lesions [4]. If the malformation is amenable to surgical treatment, the approach should always be multidisciplinary. Preoperative mapping of the anatomy usually requires a catheter angiogram, and intraoperative study of the lesion can be done non-invasively using ICG angiography. Preoperative embolization can carry significant risk compared to cranial vascular malformations because of the terminal nature and the caliber of vessels of the spinal cord [5]. Neuromonitoring can be useful especially with lesions encroaching on the cord or the conus itself. Following the pial plane into the conus is not recommended as it is likely to cause a new neurological deficit. Instead, liberating the malformation from its attachments like the filum in the conus region, followed by the careful identification of arterial feeders is key to a safer dissection. Intraoperative ICG angiograms can be very useful for that purpose. Postoperative follow-up imaging with a catheter or magnetic resonance angiogram is necessary to make sure that no residual lesion has been left behind.

## Conclusions

Arteriovenous malformations of the spinal cord are complex entities that can cause debilitating hemorrhages, especially in the young adult population. Surgical management of these lesions can be definitive but could carry significant surgical morbidity, and a systematic technical approach is required with multidisciplinary teamwork in both the intraoperative and the perioperative period. Preoperative embolization is usually not a good option because of the associated high risk of spinal infarction. Neuromonitoring for somatosensory evoked potential (SSEP), electromyogram (EMG) and motor stimulation, including anal sphincter function is required to avoid excessive manipulation of the conus. Good intraoperative vascular imaging should be available. Indocyanine green angiogram is non-invasive and can be used repeatedly. Sectioning of the filum terminale can improve mobilization of the lesion. Following a pial resection plane into the cord or conus should be avoided. Appropriate patient counseling about the possible risks and benefits of surgery is important given the potential morbidity of the procedure and of the natural history if left untreated. Follow-up vascular imaging postoperatively is necessary to confirm the obliteration of the lesion or to monitor any residual.
